# Complete Genome Sequence of Rhizobium japonicum Podophage Pasto

**DOI:** 10.1128/MRA.01442-20

**Published:** 2021-01-21

**Authors:** Nikhil Manuel, Leika Rushing, Aravind Ravindran, Heather Newkirk, Ben Burrowes, Ry Young, Carlos Gonzalez

**Affiliations:** a Department of Biochemistry and Biophysics, Texas A&M University, College Station, Texas, USA; b Department of Plant Pathology and Microbiology, Texas A&M University, College Station, Texas, USA; c Center for Phage Technology, Texas A&M University, College Station, Texas, USA; Queens College

## Abstract

Rhizobium japonicum is a Gram-negative bacterium of interest for research into nitrogen fixation in legumes. This article describes the isolation, sequencing, and annotation of R. japonicum podophage Pasto. While it shows no significant similarity to identified phages, genomic analysis indicates that Pasto may be temperate and is a novel T7-like podophage.

## ANNOUNCEMENT

Rhizobium japonicum is a Gram-negative, motile bacterium with the ability to create a symbiotic relationship with legumes (1), in which the bacterium inhabits root nodules to perform nitrogen fixation (2). R. japonicum phages can be utilized to better understand the bacterial host and possibly to facilitate understanding of the transfer of genetic material in the rhizosphere through transduction (2).

R. japonicum phage Pasto was isolated from potato root samples obtained in Olton, Texas, in August 2017. Plaques were observed on R. japonicum strain D409 (ATCC 10324) cultured on l-arabinose agar at 28°C (3). The phage DNA was isolated with a Wizard DNA cleanup kit as described before (4), and libraries were prepared with 550-bp inserts using a TruSeq Nano kit and sequenced on an Illumina MiSeq v2 system (500 bp). The 1,066,802 total reads were quality controlled and manually trimmed with FastQC (https://www.bioinformatics.babraham.ac.uk/projects/fastqc) and FastX v0.0.14 (http://hannonlab.cshl.edu/fastx_toolkit/download.html) before assembly into a single contig at 41.8× coverage with SPAdes v3.5.0 (5). The genome was closed by PCR performed off the end of the contig (forward primer, 5′-GGCAGACACACGAGAGATAAA-3′; reverse primer, 5′-TTGTTCCGTTCGTCTTGTGTG-3′) and Sanger sequencing of the resulting product. Structural annotation was performed using GLIMMER v3 (6) and MetaGeneAnnotator v1.0 (7), and tRNAs were predicted with ARAGORN v2.36 (8). Gene functions were predicted by searching through conserved domains with InterProScan v5.33 (9) and determining sequence similarity with BLAST v2.9.0 (10) by comparison with the NCBI nonredundant, Swiss-Prot, and TrEMBL databases (11). TMHMM v2.0 was also utilized (12), and the LipoP v1.0 tool evaluated lipoylation signals (13). progressiveMauve v2.4 was used to calculate genome-wide DNA sequence similarity (14). All annotation tools were run with default parameters and accessed through the Center for Phage Technology (CPT) Galaxy/Apollo Web platform (https://cpt.tamu.edu/galaxy-pub) (15–17).

Pasto is a 42,407-bp podophage with a G+C content of 58.6%, which is lower than the average host G+C content of 64.1% (3). Genomic analysis predicted 1 tRNA and 56 protein-coding genes, yielding an overall coding density of 92.8% despite the first 1.0 kb being devoid of coding sequences (CDSs). PhageTerm (18) was used to predict the phage termini, which were identified as 271-bp direct terminal repeats. Thirty-one of the 56 genes were assigned putative functions, of which 12 appear to be similar (BLASTp or BLASTn E values of <0.001) to those encoded by Escherichia coli phage T7. This finding suggested that Pasto is a podophage, which was confirmed visually by transmission electron microscopy. Surprisingly, Pasto has many hypothetical proteins that are similar to bacterial proteins, suggesting that it is related to temperate phages despite the prevalence of genes similar to those of T7. A single tRNA gene was found embedded with an antisense orientation within a CDS encoding a hypothetical protein. This tRNA sequence may be a prophage integration site, as previously observed for a mobile genetic element in *Rhizobium* (19, 20).

### Data availability.

The Pasto genome was deposited in GenBank with accession number MT708545.1. The associated BioProject, SRA, and BioSample accession numbers are PRJNA222858, SRR11558345, and SAMN14609644, respectively.
